# Application of Zwitterions in Forward Osmosis: A Short Review

**DOI:** 10.3390/polym13040583

**Published:** 2021-02-15

**Authors:** Yu-Hsuan Chiao, Arijit Sengupta, Micah Belle Marie Yap Ang, Shu-Ting Chen, Teow Yeit Haan, Jorge Almodovar, Wei-Song Hung, S. Ranil Wickramasinghe

**Affiliations:** 1 Advanced Membrane Materials Research Center, Graduate Institute of Applied Science and Technology, National Taiwan University of Science and Technology, Taipei 10607, Taiwan; ychiao@uark.edu; 2 Department of Chemical Engineering, University of Arkansas, Fayetteville, AR 72701, USA; sc068@uark.edu (S.-T.C.); jlalmodo@uark.edu (J.A.); 3 R&D Center for Membrane Technology and Department of Chemical Engineering, Chung Yuan Christian University, Chung Li 32023, Taiwan; mbmyang@gmail.com; 4 Bhabha Atomic Research Centre, Radiochemistry Division, Mumbai 400085, India; rijitbarc@gmail.com; 5 Homi Bhabha National Institute, Mumbai 400012, India; 6 Research Centre for Sustainable Process Technology (CESPRO), Faculty of Engineering and Built Environment, Universiti Kebangsaan Malaysia, Bangi 43600, Selangor Darul Ehsan, Malaysia; yh_teow@ukm.edu.my; 7 Department of Chemical and Process Engineering, Faculty of Engineering and Built Environment, Universiti Kebangsaan Malaysia, Bangi 43600, Selangor Darul Ehsan, Malaysia

**Keywords:** zwitterion, forward osmosis (FO), draw solution, antifouling, antibacterial

## Abstract

Forward osmosis (FO) is an important desalination method to produce potable water. It was also used to treat different wastewater streams, including industrial as well as municipal wastewater. Though FO is environmentally benign, energy intensive, and highly efficient; it still suffers from four types of fouling namely: organic fouling, inorganic scaling, biofouling and colloidal fouling or a combination of these types of fouling. Membrane fouling may require simple shear force and physical cleaning for sufficient recovery of membrane performance. Severe fouling may need chemical cleaning, especially when a slimy biofilm or severe microbial colony is formed. Modification of FO membrane through introducing zwitterionic moieties on the membrane surface has been proven to enhance antifouling property. In addition, it could also significantly improve the separation efficiency and longevity of the membrane. Zwitterion moieties can also incorporate in draw solution as electrolytes in FO process. It could be in a form of a monomer or a polymer. Hence, this review comprehensively discussed several methods of inclusion of zwitterionic moieties in FO membrane. These methods include atom transfer radical polymerization (ATRP); second interfacial polymerization (SIP); coating and in situ formation. Furthermore, an attempt was made to understand the mechanism of improvement in FO performance by zwitterionic moieties. Finally, the future prospective of the application of zwitterions in FO has been discussed.

## 1. Introduction

Water scarcity has become one of the major global concerns of our time. With the exponential economic development and population growth, water resource management is important [1,2]. Membrane technology is an essential technique for industrial application in water treatment since the appearance of Loeb-Sourirajan membranes on the market in 1961 [3,4]. This is due to the great advantages of membrane technology such as continuous separation processes, low energy consumption, small footprint, ease of scale-up, and so on [5]. Various driving forces such as pressure, temperature, electrical potential, and concentration have been investigated in the past half-century. In the recent decade, forward osmosis (FO) has attracted the attention of various applications like desalination [6,7,8,9,10,11], wastewater treatment [12,13,14], food processing [7,15,16], organic solvent separation [17], power generation [18,19,20], and pharmaceutical industry [21,22].

FO is a sustainable membrane process which adopts osmotic pressure difference to be the driving force across the membrane barrier [23,24]. The permeate is driven from the low osmotic pressure feed solution (FS) side to the high osmotic pressure draw solution (DS) side. It is interesting to note that unlike the relatively high energy cost needed for a pressure-driven reverse osmosis (RO) membrane, the osmotic-driven process of FO only requires minimal electric energy usage for pumping the flow operation [25,26].Because of low hydraulic pressure in FO [27], FO has been reported to have lower fouling tendency, easier foulant removal [28,29], and higher water recovery [2,13]. However, some investigators have indicated that this may not always be the case [25]. These observations suggest that the degree of fouling is dependent on the feed properties. Nevertheless, the high FO flux reversibility advantage was also evaluated in their study where it is stated the FO is a resilient membrane [25]. FO still has a great promise for application in water treatment in the future.

Despite the minimal energy demand for FO, there are several challenges to consider in the development of FO: concentration polarization (CP), DS recovery, and fouling deposition in case of a high fouling feed stream [30]. CP can be divided into two types: internal concentration polarization (ICP) and external concentration polarization (ECP) [31]. The ECP could be mitigated by changing the operation condition such as the crossflow velocity and the spacer and module channel design, because it occurs on both side of the membrane surface [32]; by contrast, membrane support layer influences the ICP, so the solution is to modify the membrane structure such as porosity, tortuosity, and thickness to minimum the effect of ICP [30]. Electrospinning technology has been employed to be a substrate to decrease the ICP effect because of high porosity and low tortuosity properties of its unique hierarchical re-entrant textile structure [33,34]. Membranologists have been investigating the membrane formation to create vertically oriented porous substrates (VOPSs) for minimizing or eliminating the ICP effect [35]. To make practical operation feasible, DS recovery is the other challenge to FO. Elimelech and coworkers used a water-soluble mixture of NH_3_ and CO_2_ containing ammonium bicarbonate (NH_4_HCO_3_) as a thermoresponsive draw solution, which resulted in the recovery at 60 °C thereby showing its potential for development in a large-scale facility [36]. Later, different novel draw solutes have been synthesized. For instance, magnetic-responsive nanoparticles, thermo-responsive materials and stimuli-responsive polymer hydrogels [2]. Apart from that, other membrane processes like membrane distillation (MD) and nanofiltration (NF) have been investigated to regenerate the draw solution continuously [37].

Fouling mitigation is a major issue for most membrane separation applications [38,39,40,41,42]. In FO, the degree of fouling is not only related to the feed composition and characteristic of the foulant, but also the hydrodynamic conditions, DS composition, and membrane properties and orientation [43]. Typically, organic fouling, inorganic scaling and biofouling have mainly attracted extensive attention and investigation. For membranologists, the main solution to mitigate the fouling deposited on the membrane is to modify the membrane properties or structure. A wide range of antifouling materials could be used to enhance the antifouling property. Graphene oxide (GO), carbon quantum dots (CQDs), halloysite, zeolite, nanoparticles, hydrophilic monomer, or polymer, and so on were used to modify the FO membrane. Modification using these materials revealed excellent results in mass transport performance and antifouling test. Recently, the anti-biofouling of the FO membrane has been investigated and reported. Hu and coworkers mentioned that dopamine-assisted silver nanoparticles(Ag NPs) coating on the membrane surface reveals that biofilm is significantly thinner than the pristine membrane and 97% lower number of *Pseudomonas aeruginosa* attached in the Ag NPs coated FO membrane [44].

Throughout the history of developing antifouling materials, they could be divided into three generations, 2-hydroxyethyl methacrylate (HEMA)-based, PEGylated-based, and zwitterionic-based materials. HEMA-based material has rich -OH moieties to generate a tight hydration layer reducing the hydrophobic foulant approach to the layer, but their antifouling property may be lost once it contacts with complex media like human blood serum and plasma [45]. The complex physicochemical interactions between the protein and the surface can affect their fouling behavior as well. The second generation is a PEG-based or oligo (ethylene glycol) (OEG) material and it demonstrated an excellent behavior to avoid protein and cell adhesion onto the interface owing to the close hydration layer around OEG chain. The most significant disadvantage is its chemical scalability, when oxygen and transition metal ions were found in biochemical environment [46]. Both HEMA and PEG materials have shown a mediocre antifouling performance once the positively charged foulants are in contact though a good fouling resistance to the negatively charged pollute because of an electrostatic repulsion [47]. Thus, zwitterionic-based material has gradually attracted researcher’s attention, and therefore regraded as third-generation antifouling material.

**Figure 1:**
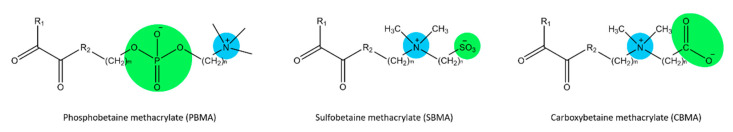
The chemical structures of antifouling zwitterionic material, PBMA, SBMA, and CBMA systems.

Zwitterionic materials are promising candidates to enhance surface hydrophilicity and augment both antifouling and antibacterial properties of the membrane because it could create a strong hydration layer through electrostatic and hydrogen bond interaction [48,49,50,51,52]. The typical zwitterionic material systems can be separated into phosphobetaine methacrylate (PBMA), sulfobetaine methacrylate (SBMA), and carboxybetaine methacrylate (CBMA) [53,54], shown in Figure 1. Similarly, they are comprised of cations and anions on the same side chain, which could prevent surface adhesion by either positive or negatively charged foulants. The zwitterionic phoshatidycholine displays anti-biofouling ability and biocompatibility to protect cell membranes, consequently, it could theoretically be used its concept to fabricate or design a related antifouling membrane or interfaces.

Zwitterionic materials have been widely studied and evolved in several membrane separation processes and explores their advantages. This review summarizes how the introduction of zwitterionic moieties can improve the antifouling characteristics of FO membrane without deterioration of the membrane performance.

## 2. Classification of Fouling Types in FO

### 2.1. Organic Fouling

Organic fouling plays a vital role in deteriorating the membrane performance during FO [20,39,55]. Humic acid, polysaccharides, colloidal particles, protein, lipid, alginate, folic acid are some common organic foulants reported in the literature. Solute agglomeration and subsequent pore blocking would result in the primary fouling of the membrane. This led to cake formation and influence in effective surface area, surface roughness and lipophilicity of the membrane surface [56,57]. This modification of the membrane surface eventually aggravates the organic fouling. Due to the salt reverse flux from DS, the salt ions can be trapped into the fouling layer. This would lead to a severe drop in water permeability and modify the hydrodynamic conditions. At extensive fouling, the phenomena highly influenced by the interaction between the foulant molecules and the fouling layer generated as a barrier on the active surface of the membrane. Divalent cations like Ca^2+^ or Mg^2+^ were also found to aggravate organic fouling, specially in the presence of humic acid by bridging the -OH and -COOH group of the same [58]. Hydrophilic naturals were reported to have higher fouling capacity compared to hydrophilic acids, while transliphic acid has higher fouling capacity than hydrophilically charged ones, whereas the hydrophilic acid has more fouling capacity than transliphic acid. Hence, the adhesion tendency of polysaccharide was reported to be three times greater than that of humic acid. The permeate flux was reduced because of adsorption of organic foulant, which can be expressed as follows:

(1) $$J=\;\frac{-\;\Delta \pi }{\eta \;R}$$

where J is the water flux, Δπ is the osmotic pressure difference between feed side and permeate side, η is the viscosity coefficient of the feed solution and R is the total resistance for organic fouling. R is a function of different factors like the intrinsic resistance of the membrane in the presence of pure water (R_i_); resistance due to concentration polarization (R_CP_); resistance due to cake formation (R_C_); resistance due to pore clogging (R_P_) and resistance due to organic foulant adsorption (R_a_). Assuming adsorption of organic foulant is the major phenomena, the water permeability can be expressed as:

(2) $$A=\;\frac{1}{R_{i}+\;R_{a}}$$

The number of interaction sites on the membrane surface plays a vital role in determining the initial rate of adsorption. If we consider the interaction sites are homogeneously distributed on the membrane surface and there is no influence on neighboring sites of interaction; then:

(3) $$r_{i}=\;\underset{i}{\sum }k_{i}\theta_{i}$$

where, r_i_ is the initial rate of interaction between organic matter and the membrane surface. k_i_ is the rate constant of the ith component and θ_i_ is the surface coverage. This model is known as the Langmuir model. If C is the concentration of the organic and K_L_ is the Langmuir adsorption coefficient, then θ can be expressed as:

(4) $$\theta =\;K_{L}\;C$$

The declination in water flux would be linearly proportional to the amount of organic substance adsorbed on the membrane surface:

(5) $$J_{0}-J=\;k_{L}\left(C_{0}-C\right)V$$

$The\;k_{L}$ is proportionality constant and V is the volume of permeate. The concentration of organic adsorbed on the membrane surface can be expressed as:

(6) $$C=\;C_{0}-\;\frac{J_{0}-\;J}{k_{L}V}$$

(7) $$\theta =\;K_{L}\;\left(C_{0}-\;\frac{J_{0}-J}{k_{L}V}\right)$$

(8) $$r=\;\frac{dJ}{dt}=kK_{L}\left(C_{0}-\;\frac{J_{0}-J}{k_{L}V}\right)$$

Integrating after putting the boundary condition; J = J_0_, when t = 0, the equation becomes:

(9) $$J=\;J_{0}+\;k_{L}VC_{0}\;\left(e^{\frac{kK_{L}}{k_{L}V}t}-1\right)$$

The natural organic matter can broadly be divided into two groups depending on their origin as follows:

(1) Autochthonous: They are obtained from extracellular macromolecules from microorganism and carbon fixation by algae and aquatic plants.
(2) Allochthones: They are obtained from the decayed parts of plants and animals.

**Figure 2:**
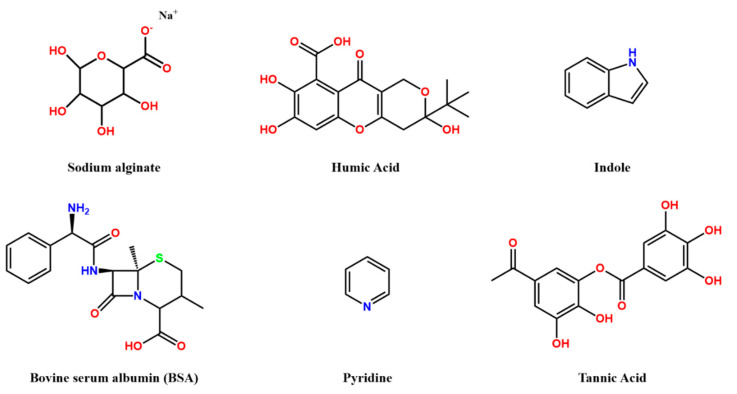
Simplified chemical structures for some of the organic foulants.

The structures of some common organic foulants are shown in Figure 2**.** Organic foulants with large size/molecular weight are reported to cause reversible fouling, whereas organic foulants of small molecular weight lead to irreversible fouling and are difficult to remove from the membrane surface. 

### 2.2. Inorganic Fouling

Inorganic fouling occurs when the solubility of sparingly soluble inorganic materials (SiO_2_, CaCO_3_, CaSO_4_, MgSO_4_, BaSO_4_, etc.) present in the feed solution exceed their solubility limit near the membrane surface [59,60,61,62]. The reverse diffusion of bivalent salt ions from the draw solution was also reported to enhance the cake formation by bridging and interacting with other substances present in the feed solution. Out of the different foulants responsible for inorganic scaling, silica—with a ~120 mg·L^−1^ solubility limit—is one of the most common materials. Beyond this concentration, severe inorganic scaling is reported. This silica deposition ultimately leads to the formation of silica gel films on the membrane surface because of polymerization resulting in deterioration of membrane performance. The nature of the membrane material was also found to significantly influence the inorganic scaling in FO process. The stronger adhesion force between silica gel and the polyacetate membrane surface resulted in stronger inorganic scaling compared to that observed on a cellulose acetate membrane surface [59]. A comparative evaluation of inorganic scaling on thin film composite membrane having multiple -COOH groups vis-a-vis cellulose acetate membrane was reported. The dipolar -COOH functional groups resulted in dipolar interactions leading to silica deposition on membrane surface. In the next step, monosilisic acid started interacting resulting in -Si-O- bond and eventually leading to polymerization, resulting to formation of silica gel. Severe gypsum scaling was reported on polyamide membrane compared to cellulose acetate (CA) and cellulose triacetate (CTA) membrane materials in FO operation [59].

### 2.3. Biofouling

Biofouling in FO is the most complicated fouling process and is the most difficult to control. Biofouling is the deposition of a microbial community on a membrane surface followed by the formation of biofilms [63,64,65,66]. This is a multicellular architecture of live and dead cells in a self-produced gel like extracellular polymeric substances mainly containing proteins and polysaccharides. Because of the stronger adhesion, simple shear force, and physical cleaning, even the use of oxidizing substances may not be effective for the recovery of FO membranes once it undergoes extensive biofouling. This biofouling can be explained as a combination of two-step phenomena:

(1) The transportation of bacterial cells near the membrane-feed solution interface and attachment of bacterial cells on the membrane surface. This step is reversible in nature and is mainly governed by van der Waal’s forces.
(2) In the subsequent step, there is a formation of a bacterial colony leading to a stronger interaction with membrane materials, formation of biofilms, and eventually modification of the membrane surface properties. This step is irreversible in nature.

FO biofilms are more open structures compared to those in RO mode. Most of the bacterial cell walls possess plenty of phosphoryls and carboxylic acid functional groups. As a result, the bacterial cell wall is mostly negatively charged. Therefore, a membrane surface with negative zeta potential is the ideal choice to avoid bacterial attachment. Additionally, a heterogeneous polyamide FO membrane surface was reported to be more susceptible to biofouling compared to cellulose acetate due to its hydrophobicity [67]. Biofouling can also lead to pore blocking and hence initiate other types of fouling, like inorganic scaling. Chemical cleaning with chlorine is one of the unique methods for the addressing biofouling, which can further be improved by high crossflow velocity.

### 2.4. Colloidal Fouling

Colloidal particles are intermediate in size. They are neither truly dissolved solid nor suspended solids. They are mainly contributed by clay, corrosion products, silica, etc. [68,69,70]. This colloidal fouling could lead to an increase in cake-enhanced osmotic pressure resulting in reduction of driving force and the permeate flux. The colloidal fouling can affect the FO performance mainly in two ways:

(1) Cake layer hydraulic resistance
(2) Cake enhanced osmotic pressure (CEOP)

CEOP (Δπ*) can be expressed as follows:

(10) $$\Delta \pi^{*}=2\mathrm{RT}\Phi R_{o}C_{b}\exp\left(\frac{J_{0}}{k^{*}}\right)=2\mathrm{RT}\Phi C_{b}{\mathrm{CP}}^{*}$$

where R is the gas constant, T is temperature (K), Φ indicates the molar osmotic coefficient, R_o_ indicates the salt rejection, C_b_ indicates the bulk salt concentration, CP^*^ indicates the cake-enhanced concentration polarization, J_0_ indicates the initial flux, k^*^ indicates the cake-hindered mass transfer coefficient. Initially, cake layer formation and cake layer hydraulic resistance play a pivotal role in colloidal fouling. However, with time, CEOP becomes the major contributing factor towards hydraulic fouling.

## 3. Zwitterionic Membrane

To date, several strategies have been used to functionalize surfaces/membranes. Zwitterionic functionalized membrane modification could typically be divided into four methodologies: in-situ, second interfacial polymerization, coating, and atom transfer radical polymerization ATRP.

### 3.1. In-Situ

**Figure 3:**
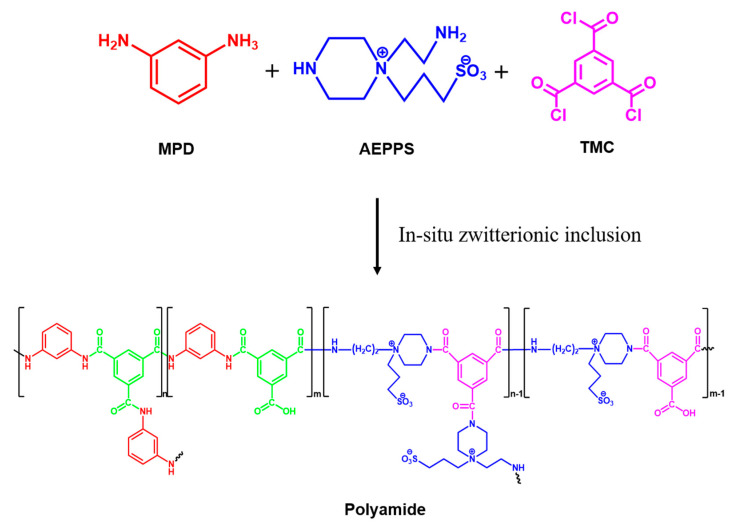
The schematic diagram for in-situ inclusion of zwitterionic moieties in FO membrane (reproduced from Chiao et al. [13]).

Additives are blended in a casting solution or an interfacial polymerization precursor solution, which is an easy method to fabricate a functionalized FO membrane. Thus, one single step “in-situ” modification has attracted researchers’ and membrane manufacturers’ attention [71]. Ismail’s group [72] synthesized a zwitterionic polymer, poly[3-(N-2-methacryloylxyethyl-N,N-dimethyl)ammonatopropanesulfonate] (PMAPS) and blended it with polyethersulfone (PES) to fabricate a substrate to form a TFC-selective layer following interfacial polymerization. With a higher PMAPS content, the flux could increase from 12.54 to 15.79 L·m^−2^h^−1^ (1% PMAPS) using 2 M NaCl draw solution due to the enhancement of hydrophilicity. However, overloaded PMAPS led to a significant reverse solute flux jumping from 3.56 gMH (0% PMAPS) to 24.52 gMH (5% PMAPS) because of bigger pore size support membrane that causes the PA layer to not fully cover the membrane surface. To test the antifouling behavior, an oily wastewater treatment was performed in the active layer facing the draw solution (AL-DS) mode zwitterionic support layer facing to the feed stream. Owing to the dense selective layer, the oil rejection was maintained at 99%. Furthermore, a four cycles long-term test along with a high oil concentration were used to evaluate the antifouling performance. After increasing the concentration, the flux decline was severe for the control membrane. The normalized water flux dropped at 51.2% for 10,000 ppm oil, which was higher than 1% PMAPS-PES ~34.8%. This was because the SO_3_ forms a hydration layer with water molecules, which prevent oil droplet adhesion onto the membrane support. Chiao et al. [13] synthesized a zwitterionic diamine monomer, N-aminoethyl piperazinepropane sulfonate (AEPPS), which could mix in the interfacial polymerization precursor aqueous solution and react with the organic phase precursor TMC to form a cross-linked polyamide layer on the membrane. The main purpose to this resulting membrane was to augment the antifouling ability and enhance the membrane performance. The water flux was gradually increased as more AEPPS was involved into the reaction, from ~10 L·m^−2^h^−1^ (0% AEPPS) to ~17 L·m^−2^h^−1^ (50% AEPPS). The 30% had the lowest specific slat flux ~0.4 g/L. Because of the tight hydration layer, improved antifouling properties were expected. Thus, the static and dynamic fouling were used to challenge and prove it. The model protein foulants BSA and lysozyme solution were used to determine the adsorption ability on the membrane surface. At pH 7, BSA exists as negatively charge protein, whereas lysozyme exists as positively charged protein (their isoelectric point is as follow: BSA= pH 4.7, and lysozyme= pH 11.0) [47]. With more AEPPS participating the reaction, the surface adsorption capacity was reduced either using BSA or lysozyme solution. In the dynamic fouling test, 800 ppm sodium alginate was used to be a model organic foulant which can form a gel layer and decrease the water permeability. 50% AEPPS membrane could maintain normalized flux more than 75% after 700 min of FO mode operation, which was higher than 0% AEPPS membrane 60%. This resulting zwitterionic membrane not only enhanced the water flux, but also the static and dynamic antifouling ability due to the surface hydrophilicity formed by the zwitterionic tight hydration layer. However, the selectivity structure or crosslinking degree is changing. There is a possibility to lose the selectivity ability and increase the solute reverse flux even the permeability could be enhanced. The amount of additive must be added carefully, as it plays an important role to in-situ modification [14]. Figure 3 schematically presents the in-situ inclusion of zwitterionic moieties in a FO membrane.

### 3.2. Second Interfacial Polymerization (SIP)

**Figure 4:**
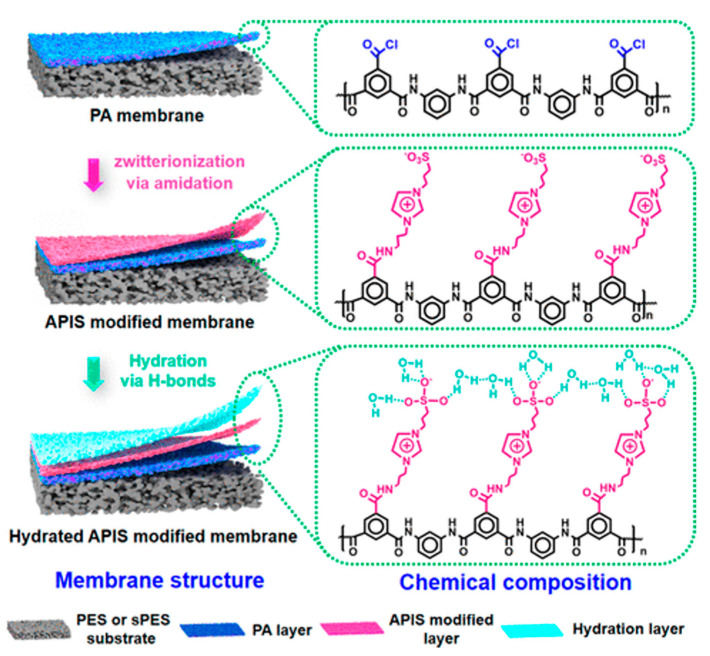
Schematics of 2nd interfacial polymerization for inclusion of zwitterionic moieties on FO membrane (reproduced from Chen et al. [76]).

Second interfacial polymerization is regarded as an in-situ modification technique [73], however, the SIP route does not obviously affect the structure of the PA selectivity layer. The PA layer is functionalized by additives with a covalent bond to cross-link with the typical PA structure. Thus, it is discussed independently and separately from in-situ modification. Chiao et al. [14] synthesized the zwitterionic monomer AEPPS from 1-(2-aminoethyl)piperazine (AEP) and 1,3-propanesultone via a ring-opening reaction. The amine group on AEPPS reacted with the chloride acid group on the TMC after generating a typical selectivity structure PA. The resulting functionalized PA layer (PAZ) enhanced the hydrophilicity significantly from ~80° to ~15°, because of the tightly-bound water layer around the zwitterionic moieties. Meanwhile, the super-oleophobicity was found for PAZ (~160°) in the underwater oil contact angle test. In general, the superoleophobic surface has the value above 150° [74]. From the morphology, the mitigated nodule structure and smoother surface was found after SIP, which was beneficial for the antifouling property. The water flux was improved from 11.48 to 18.91 L·m^−2^h^−1^ using 1 M NaCl solution as a draw solution in FO mode. Through the SIP, the structure parameter (S) was reduced about 43% compared to the controlled membrane because of the superhydrophilicity of the zwitterionic moieties. Meanwhile, the antibacterial property of the zwitterionic functionalized membrane significantly improved as expected [75], where the amount of attached *E. coli* was reduced from 4810 cell/mm^2^ to 352 cell/mm^2^. Besides, the protein foulants BSA, and lysozyme was used to investigate the performance. At pH 7.4, the protein surface charge of BSA and lysozyme were −22 and +3.5 mV; consequently, the positive and negative charge could be a good model foulant to challenge the zwitterionic surface. Modified membrane PAZ demonstrated an excellent antifouling behavior attributed to the surface charge and roughness. Apart from a static test, a dynamic fouling test using simulated city wastewater, which is a common model solution to simulate the polysaccharides present in real wastewater, was performed too. The normalized flux of the modified membrane could be maintained above 90% before achieving 50% recovery. The real produced water was filtered with a PAZ membrane. In the commercial aspect, reduction of wastewater discharge is an enormous challenge when using the horizontal drilling and hydraulic fracturing technique. It consists of oil and grease, salt, surfactants and so on; thus, regulators strictly define the allowed discharge locations. Chen et al. designed a zwitterionic amine monomer, (1-(3-aminopropyl) imidazole) propane sulfonate (APIS) and grew it on a PA layer. The surface hydrophilicity decreased significantly from ~70° (PA-PES) to ~40°; meanwhile, 101% higher water flux than the unmodified membrane [76]. Figure 4 schematically presents the inclusion of zwitterionic moieties by 2nd interfacial polymerization.

### 3.3. Coating

Zwitterion functionalized carbon nanotubes (Z-CNTs) coated onto a commercial Aquaporin Inside^®^ FO thin film composite (TFC) membrane have been reported by Zou et al. [77]. They obtained BSF mitigation through zwitterion-induced repulsive electrostatic interaction along with the carbon nanotube-induced steric interaction. The presence of extended repulsive interaction in presence of low ionic strength liquid, the coating of zwitterion-functionalized CNT on the active side of the membrane was found to provide better mitigation of reverse salt flow in active layer feed facing mode. They have reported a reduction in specific reverse salt flow of 84% for using NH_4_H_2_PO_4_ as draw solution, 75% for (NH_4_)_2_HPO_4_ as draw solution, 71% for NH_4_Cl as draw solution using an optimum coating density of 0.97 g m^−2^. After 12 days operation of actual wastewater, only marginal reduction in water flux (~15% reduction) was reported compared to that of virgin membrane (~55% reduction). Simple physical flushing was found to be effective for the removal of any foulants adsorbed on the membrane surface.

**Figure 5:**
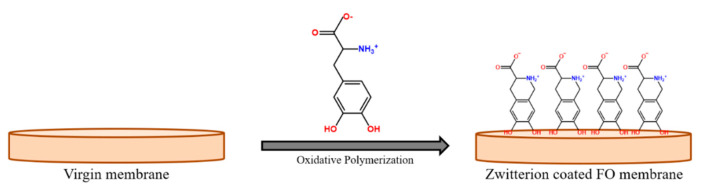
The schematic of formation of zwitterionic polymer coated FO membrane [78].

Neguyen et al. [78] have reported the surface modification of commercially available flat sheet cellulose triacetate FO membrane by coating of a zwitterion (polyamino acid 3-(3,4-dihydroxyphenyl)-L-alanine (L-DOPA)) on the porous side of the membrane, to improve the antifouling characteristics of membrane in pressure retarded osmosis mode. As indicated earlier, the coating of zwitterions on membrane surface would naturally enhance the hydrophilicity of the membrane surface and the repulsive electrostatic interaction provided by the cationic as well as anionic moieties of zwitterions lead to the reduction in fouling propensity. The L-DOPA-coated membranes showed 30 % less fouling probability compared to a virgin membrane after 12 h of FO operation in PRO mode using alginic acid sodium salt and CaCl_2_ as feed solution. Almost 90% recovery of the L-DOPA-coated membrane was reported by simple hydraulic water treatment. Figure 5 schematically present coating of zwitterions on FO membranes.

### 3.4. Atom Transfer Radical Polymerization (ATRP)

**Figure 6:**
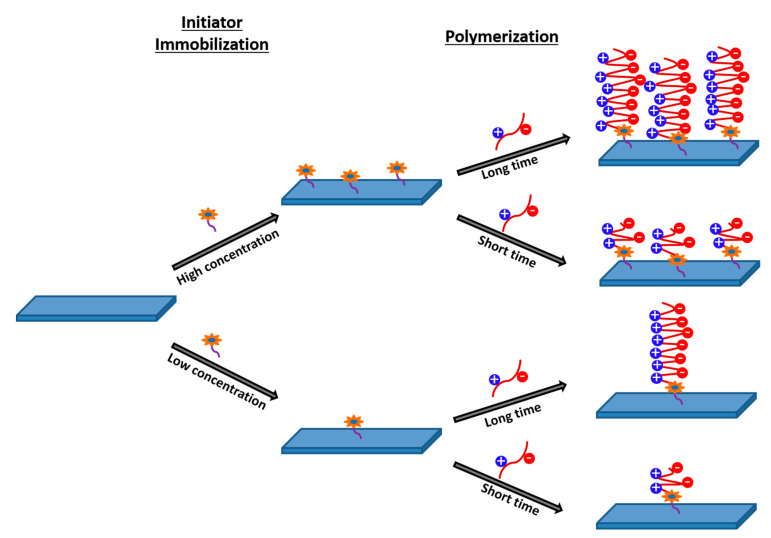
The schematic presentation for ATRP to graft zwitterionic brush.

ATRP is a technique for control the grafting of zwitterions on the membrane surface. The first step is to immobilize the initiator, the site for polymer growth. The second step involves the controlled polymerization of the zwitterionic monomer, mainly utilizing the equilibrium of Cu^2+^/Cu^1+^ salt (Figure 6) [79,80,81]. The duration of the initiator immobilization step influences the grafting density, while the length of the zwitterionic polymeric chain was influenced by the duration of the 2nd step of ATRP. Liu et al. [82] reported a comprehensive investigation in understanding the efficacy of the poly(sulfobetaine methacrylate) grafted thin-film composite membrane vis-a-vis a dense layer of hydrophilic silica nanoparticle impregnated thin-film composite FO membrane towards antifouling characteristics. The nanoparticle-impregnated membrane was found to be ineffective in shielding the surface carboxylic acid groups resulting in an electrostatic attractive interaction with protein molecules and Ca^2+^ ion present as foulant. However, the presence of a polymeric zwitterionic chain on the membrane surface not only enhanced the surface hydrophilicity, but also improved the hydration layer of zwitterionic polymeric brush provided a large physical as well as energetic barrier to shield the carboxylic acid functional group. Thus, neither protein molecules nor inorganic/organic moieties can be adsorbed on the membrane surface. The microorganism adsorption test was performed using *E coli*. A significant number of *E. coli* cells (1.44 ± 0.30 × 10^5^ cells/cm^2^) was found to grow in presence of virgin pristine thin film composite membrane. However, zwitterionic polymer grafted TFC membrane led to a reduction in 96% of *E. coli* cell counts, revealing the antimicrobial characteristics of the same.

Poly [2-(methacryloyloxy)-ethyl] dimethyl-(3-sulfopropyl) ammonium hydroxide (PSBMA) was grafted on a commercial pristine FO membrane. It was found to produce a zwitterionic TFC membrane having enhanced hydrophilicity, reduced surface charge and surface roughness [83]. PSBMA-grafted TFC membrane was reported to have a narrower adhesion force distribution, ca, −0.07 ± 0.15 mN/m, revealing a 92% reduction compared to virgin membrane. The effective shielding of carboxylic acid groups resulted in the prevention of Ca^2+^-induced fouling. Additionally, the swelling of the zwitterionic polymeric chain resulted in a further reduction of the adhesive force because of the increase of distance between the solid-water interface and the membrane surface. The protein adsorption was monitored using a FITC-BSA model by fluorescence spectroscopy. The highly intense fluorescent membrane surface for virgin membrane was attributed to the BSA adsorption, which led to almost no fluorescent membrane surface after zwitterionic polymeric brush grafting. This indicated the antiprotein adsorption characteristics of the membrane due to the presence of zwitterions. The zwitterionic modification was claimed to result in an almost 90% reduction in colony forming units (CFUs) against *E coli* bacteria. Static and dynamic fouling experiments confirmed that the presence of zwitterions on the membrane surface not only improved the hydrophilicity of the surface resulting in an enhancement in membrane permeability, but also extensively improved the antifouling characteristics of the membrane towards inorganic scaling, protein adsorption, and even bacterial/biofilm growth.

Zhang et al. [84] investigated different cleaning approaches for FO membranes having zwitterionic poly-(sulfobetaine methacrylate) brushes prepared by ATRP subjected to crude oil and anionic polyacrylamide as model fouling solution for treatment of polymer flooding-produced water. They reported a significant enhancement in irreversible fouling with increasing relative concentration of crude oil in the feed solution. Five different cleaning protocols have been adopted as follows:

(1) Simple hydraulic washing: Feed: DI water; Draw solution: DI water; pH 7
(2) Osmotic backwashing: Feed: 2 M NaCl, Draw solution: DI water; pH 7
(3) Acid cleaning: Feed: 0.1% Citric acid; Draw solution: DI water; pH 3
(4) Alkaline cleaning: Feed: 0.1% NaOH, Draw solution: DI water; pH 12
(5) Surfactant cleaning: Feed: 0.1% SDBS solution; Draw solution: DI water; pH 9

Simple hydraulic washing was found to be an inefficient procedure, while an excellent cleaning efficiency and chemical stability was reported for poly(sulfobetaine methacrylate)-grafted FO membranes towards the surfactant cleaning agents. However, poor chemical stability was reported for high pH alkali cleaning agents. A gradual formation of irreversible fouling on PSBMA grafted membrane was reported and attributed to the insufficient capability of the DI water for removal of the accumulated oil foulants from the membrane surface. The synergistic effect of the NaCl solubilization and water back-flushing was found to be responsible for the increase in cleaning efficiency of the foulants accumulated on the membrane surface. The SDBS cleaning protocol was demonstrated to be the most efficient protocol to remove foulants from the surface as this surfactant formed a macromolecular micellar agglomeration to dissolve oil, fat protein and other lyophilic foulants in water.

## 4. Draw Solution

The draw solution in FO exhibits a very important role in improving the water permeability and the back-diffusion of the components of the draw solution. Though conventionally, inorganic salts solution (e.g., NaCl, MgSO_4_. etc.) was used as a draw solution in FO process, a vast amount of research has been carried out on the nature of draw solutions and their effect on FO performance. Application of organic molecules as draw solution was reported to induce advantageous properties. The size being larger compared to inorganic cations, the rate of back diffusion is slower [85]. Reports were also available on using highly volatile diethyl ether as a draw solution to facilitate the recovery of the permeate. Polyelectrolytes have also been exploited as a draw solution to reduce the reverse solution flux. However, only a marginal improvement in FO performance was noticed compared to the presence of organic molecules as draw solution. The formation of a cross-linking network with water molecules, and the stimuli-imposed dehydration characteristic of hydrogel have been exploited to use hydrogels in the draw solution. Reduction in osmotic pressure because of the Gibb’s-Donnan effect limits the application of hydrogel. Using stimuli responsive polymer (mainly temperature, chemical environment, pH, etc.) as draw solution has also drawn the interest of researchers.

Ju and Kang have reported the application of zwitterionic polysulfobetaine homopolymers as draw solution in FO performance [85]. The upper critical solution temperature for polysulfobetaine- water was reported to be 40 °C. This implies that if the solution temperature is less than that of 40 °C, the ion-ion interaction between the zwitterionic moieties is stronger than that of zwitterion-water interaction resulting in zwitterionic agglomeration and phasing out from aqueous medium, whereas the reverse is true for the solution temperature more than 40 °C, leading to homogeneous zwitterion-water solution. Thus, after FO operation, the recovery of water from the polymeric zwitterionic aqueous solution was achieved by the temperature response of the polymer. As the concentration of the polymeric zwitterionic brush was enhanced from 5 wt% to 10 to 15 to 20 wt%; the water flux values followed the order, 0.92, 1.39, 2.30, 3.22 L·m^−2^h^−1^, respectively. The reverse solute flux was reported to increase with the increase in draw solution concentration as follows: 0.27 gMH at 5 wt%, 0.29 gMH at 10 wt%, 0.35 gMH at 15 wt%, and 0.36 gMH at 20 wt%. Their investigation provided a new dimension in research on draw solute.

Lutchmia et al. [86] reported a comparative evaluation on the application of different zwitterions (glycine, L-proline, L-valine, L-glutamine and glycine betaine) as draw solution in FO process vis-a-vis aqueous NaCl solution, the conventional draw solution in wastewater reclamation. The investigation revealed that glycine, L-proline and glycine betaine exhibited comparable water fluxes to the conventional draw solution, NaCl and the reported water flux was 5 L·m^−2^h^−1^. However, a significantly low solute loss was reported by using these zwitterions in draw solution (glycine: 2.13 g/m^2^h; L-proline: 1.377 g/m^2^h; glycine betaine: 0.967 g/m^2^h; and NaCl: 3.26 g/m^2^h).

The physicochemical parameters of zwitterions as draw solution, which significantly influence the FO operation are as follows:

(1) Osmotic pressure
(2) Charge
(3) Polarity
(4) Molecular weight

### Osmotic Pressure:

The diluted internal concentration polarization for conventional NaCl solutions was reported to be less than that of zwitterions. Zwitterionic glycine showed severe diluted internal concentration polarization compared to proline. The diffusivity and the viscosity of the draw solution significantly influenced the diluted internal concentration polarization. Highly soluble draw solutes were found to enhance the water permeability with enhancement in osmotic pressure difference.

### Charge:

Zwitterions possess dipoles because of the separation of the positive and negative charge in the same moiety. In the case of amino acids, the zwitterionic form and its concentration depend on the pH of the medium as it is significantly influenced by pKa of the materials. According to the Henderson-Hasselbalch equation, the pKa and pH are quantitatively related as follows:

(11) $$\mathrm{pH}=\mathrm{pKa}+Log_{10}\;\left(\frac{\left[Dissociated\;form\right]}{\left[Undissociated\;form\right]}\right)$$

### Polarity:

The partitioning of the zwitterions in water vs the membrane materials played a significant role in determining the (J_s_/J_v_) flux ratio. As the hydrophobicity of the zwitterion decrease, the J_s_/J_v_ value was also reported to decrease. The polarity of the zwitterions was reported to influence their rejection.

### Molecular Weight:

**Figure 7:**
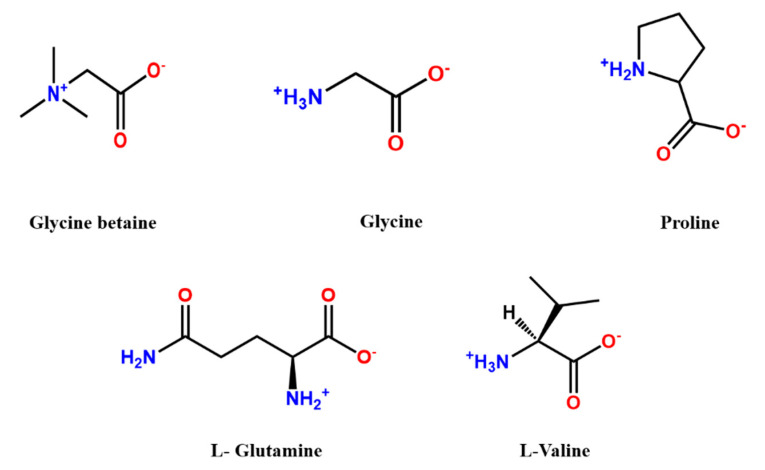
Chemical structures of some of the zwitterions used as draw solution in FO.

Because of steric hindrance, which is also called the sieving effect, the molecular weight of the zwitterions in the draw solution highly influences the reverse solute flux as discussed earlier. The diffusion coefficient of the zwitterions through the membrane pores depends on the molecular weight of the zwitterions. The molecular weight followed the trend: glutamine > glycine betaine > NaCl resulted in the trend in J_s_/J_v_ ratio NaCl > glycine betaine > glutamine. The molecular weight followed the trend: glutamine > valine ~ proline > glycine > NaCl resulting in a diffusion coefficient trend: NaCl > glycine > proline > valine > glutamine. Figure 7 shows the chemical structures for some zwitterions used as draw solutions.

## 5. Perspectives

The application of zwitterions as a draw solution has opened up a new domain of research, however, there is always a tradeoff between the osmotic pressure difference created by the draw solute and the recovery of the permeated products from the draw solution side. The future direction of research in this topic is to effectively overcome this tradeoff. Zwitterionic polymers in combination with their external stimuli response could resolve the issue. In view of that, some literature is available where temperature responsive polyzwitterionic moieties have been utilized [85]. The zwitterionic moieties are responsible for creating an osmotic pressure difference between the feed and permeate side, the driving force for FO. Subsequently, the temperature of the system is allowed to raise. The rise in temperature results in a conformational change from an extended structure to a globule structure of the zwitterionic polymers. As a result, at elevated temperature, the polymer phases out from the system and hence leads to an almost complete recovery of the permeate. Similarly, magnetic responsive zwitterionic polymers can also be another ideal choice. The zwitterionic moieties will impose an osmotic pressure difference, creating driving force for FO. After the FO process, the polymers could be separated from the draw solution by means of magnetic interactions.

For the incorporation of zwitterionic moieties in FO membranes, the distribution of zwitterionic moieties on the membrane surface should be highly homogeneous and there should be some control of the grafting density and the length of the polymers. The stability of such layer on the virgin membrane should be another aspect consider. Instead of considering physical interaction to attach the zwitterionic moieties on membrane, the chemical interaction would exhibit better stability for the attachment.

## 6. Conclusions

The main applications of zwitterions in FO processes can broadly be classified into two categories: inclusion in membrane materials and inclusion in draw solutions. The presence of positive as well as negative charges on zwitterions induces an electrostatic repulsive interaction when placed on a FO membrane surface towards the foulant present in feed solution. This not only reduces the initial microbial adhesion, inorganic scaling, but also drastically reduces bacterial colony growth. The presence of zwitterions on the FO membrane surface enhances the surface hydrophilicity and surface zeta potential compared to the virgin membrane. This enhancement in surface hydrophilicity along with the formation of an aqueous layer on the FO membrane surface resists the organic molecules to have a close proximity to feed/membrane interface. This interaction leads to a drastic reduction in organic fouling on the membrane surface. Most bacterial cell walls have a negatively charge, hence when exposed to the anionic moieties of zwitterions, there is a repulsive electrostatic interaction, resulting in a reduction in bacterial adhesion.

On the other hand, zwitterionic inclusion in draw solutions reduces the reverse solute flux compared to conventional brine solutions where inorganic salts are used. The polarity, molecular weight, and charge of zwitterions in the draw solution are the important factors controlling the reverse salt effect. Small membrane pores and large good water-soluble zwitterions are the ideal choice for FO performance.

## Abbreviations

A	Water permeability
AEP	1-(2-Aminoethyl) piperazine
AEPPS	N-aminoethyl piperazinepropane sulfonate
Ag NPs	Silver nanoparticles
AL-DS	Active layer facing the draw solution
APIS	(1-(3-Aminopropyl) imidazole) propane sulfonate
ATRP	Atom transfer radical polymerization
BSA	Bovine serum albumin
CA	Cellulose acetate
C_b_	Bulk salt concentration
CBMA	Carboxybetaine methacrylate
CEOP	Cake enhanced osmotic pressure
CFU	Colony forming unit
CP	Concentration polarization
CP^*^	Cake-enhanced concentration polarization
CQDs	Carbon quantum dots
CTA	Cellulose triacetate
DS	Draw solution
ECP	External concentration polarization
FO	Forward osmosis
FS	Feed solution
GO	Graphene oxide
HEMA	2-Hydroxyethyl methacrylate
ICP	Internal concentration polarization
J	Water flux
J_0_	Initial flux
k^*^	Cake-hindered mass transfer coefficient
k_i_	Rate constant of the ith component
K_L_	Langmuir adsorption coefficient
L-DOPA	Polyamino acid 3-(3,4-dihydroxyphenyl)-L-alanine
MD	Membrane distillation
NF	Nanofiltration
OEG	Oligo(ethylene glycol)
PBMA	Phosphobetaine methacrylate
PES	Polyethersulfone
PMAPS	Poly[3-(N-2-methacryloylxyethyl-N,N-dimethyl)-ammonatopropanesulfonate]
PSBMA	Poly [2-(methacryloyloxy)-ethyl]dimethyl-(3-sulfopropyl)ammonium hydroxide
R_a_	Resistance due to organic foulant adsorption
R_C_	Resistance due to cake formation
R_CP_	Resistance due to concentration polarization
R_i_	Intrinsic resistance of the membrane in presence of pure water
r_i_	Initial rate of interaction between organic matter and the membrane surface
RO	Reverse osmosis
R_o_	Salt rejection
SIP	Second interfacial polymerization
T	Temperature (K)
TFC	Thin film composite
VOPSs	Vertically oriented porous substrates
Z-CNTs	Zwitterion functionalized carbon nanotubes
Φ	Molar osmotic coefficient
Δπ	Osmotic pressure difference
η	Viscosity coefficient
